# The unfolded protein response genes in human osteoarthritic chondrocytes: PERK emerges as a potential therapeutic target

**DOI:** 10.1186/s13075-016-1070-6

**Published:** 2016-07-19

**Authors:** Ying-Hua Li, Ginette Tardif, David Hum, Mohit Kapoor, Hassan Fahmi, Jean-Pierre Pelletier, Johanne Martel-Pelletier

**Affiliations:** Osteoarthritis Research Unit, University of Montreal Hospital Research Centre (CRCHUM), 900 Saint-Denis, R11.412B, Montreal, QC H2X 0A9 Canada; Division of Genetics and Development, Toronto Western Research Institute, University Health Network (UHN), Toronto, ON Canada; Department of Surgery, University of Toronto, Toronto, ON Canada

**Keywords:** PERK, Osteoarthritis, Chondrocyte, ER stress, Unfolded protein response

## Abstract

**Background:**

The unfolded protein response (UPR) is activated following an endoplasmic reticulum (ER) stress. The aim of this study was to investigate the global expression of UPR genes in human OA chondrocytes in induced (I)-UPR conditions, and to explore the regulation and role of the UPR genes in homeostatic (H)-UPR conditions in human normal and OA chondrocytes.

**Methods:**

Gene expression was determined by PCR array and qPCR. Protein production in cartilage was determined by immunohistochemistry, gene silencing by specific siRNAs, and gene regulation by treating chondrocytes with cytokines and growth factors associated with cartilage pathobiology.

**Results:**

Several UPR genes, among them ERN1, PERK, and CREB3L2 were downregulated in OA compared to normal chondrocytes at both the mRNA and protein levels, but the ER stress response triggered by thapsigargin or tunicamycin treatment was similar in normal and OA chondrocytes. The activation of ER stress sensors (phosphorylated PERK, cleavage of ATF6B, and the spliced mRNA forms of XBP1) was not significantly increased in OA chondrocytes/cartilage. PDGF-BB and IL-6 significantly downregulated the expression of ERN1, PERK, and CREB3L2, but not that of ATF6B. Silencing experiments done under conditions of no ER stress (physiological conditions) revealed that decreasing ERN1 expression led to decreased COL2a1, MMP-13, ADAMTS4 and ADAMTS5 expression, while decreasing CREB3L2 and ATF6B led to decreased ADAMTS5 and ADAMTS4 expression, respectively. Importantly, the downregulation of PERK expression increased COL1a1 and suppressed COL2a1 expression.

**Conclusions:**

Although the level of ER stress is not significantly increased in OA chondrocytes, these cells respond strongly to an acute ER stress despite the decreased expression of ERN1, PERK, and CREB3L2. Emerging findings revealed for the first time that these genes play a role in cartilage biology in conditions where an acute ER stress response is not triggered and OA is not characterized by an overall basal activation of the ER stress response. Importantly, these findings identify PERK as a potential target for new OA treatment avenues.

**Electronic supplementary material:**

The online version of this article (doi:10.1186/s13075-016-1070-6) contains supplementary material, which is available to authorized users.

## Background

Osteoarthritis (OA) is a progressive disease of the joints resulting in the degradation of articular cartilage. Several signalling pathways, transcription factors, and cytokines contribute to the progression of this disease, and it is likely that other molecules will be discovered as well, as more is yet to be learned to fully understand this pathology.

The endoplasmic reticulum (ER) stress response has attracted attention as a new area of research in OA cartilage biology. ER stress can be triggered by oxidative stress, heat shock, hypoxia, expression of mutant proteins and drugs such as thapsigargin (Tg), which disrupts ER calcium ion balance [1], and tunicamycin (Tm), which inhibits N-linked glycosylation [2]. The cellular response, known as the unfolded protein response (UPR), serves to counter the stresses that occur by the presence of misfolded proteins in the ER in an attempt to restore homeostasis [3–8]. Three major families of ER stress sensors have been identified in mammalian cells: the endoplasmic reticulum to nucleus 1 (ERN1, also called IRE1), PRKR-like endoplasmic reticulum kinase (PERK, or EIF2AK3), and activating transcription factor 6 (ATF6). The ATF6 family comprises several members, among them ATF6B, CAMP-responsive element-binding protein 3-like 2 (CREB3L2), and old astrocyte specifically induced substance (OASIS), which are involved in different cellular processes or expressed preferentially depending on cell type [9, 10]. As an example, CREB3L2 has been identified as a transcription factor expressed strongly in chondrocytes [11]. Accumulation of unfolded proteins in the ER initiates the activation of ERN1, PERK, and ATF6, leading to the upregulation of several genes such as the glucose-regulated protein 78 (GRP78 or BiP or HSPA5), a chaperone that binds preferentially to the unfolded proteins.

Accumulative evidence has implicated UPR proteins in physiological processes beyond the control of protein folding [7]. In those instances, ERN1, PERK, and ATF6 can be activated without the presence of misfolded proteins and the dissociation of GRP78, and the final outcome is different from that triggered by the ER stress [12, 13].

Drug discovery efforts have recently identified the UPR proteins as therapeutic targets to treat several diseases [14]. However, although the expression and roles of some individual UPR genes have been investigated in OA [15, 16], the global response of chondrocytes to ER stress has not been looked at. The present study had two major aims. The first one was the evaluation of the global expression of the UPR genes in normal and OA chondrocytes in response to an induced ER stress (I-UPR conditions) in order to assess their impact during OA. Importantly, our second aim was to examine how the deregulation of the major representative ER stress sensors could affect cartilage metabolism under conditions where an ER stress is not induced (homeostatic or H-UPR).

## Methods

### Specimen selection

Human normal cartilage was obtained from femoral condyles and tibial plateaus from individuals within 12 hours of death (n = 18, 60 ± 19 years [mean age ± standard deviation (SD)]) and OA cartilage from patients undergoing total knee arthroplasty (n = 42, 69 ± 9 years). Human normal individuals had no history of joint disease and died of causes unrelated to arthritic diseases. The cartilage was examined macroscopically and microscopically to ensure that only normal tissue was used. All OA patients had been evaluated by a certified rheumatologist and diagnosed as having OA according to the American College of Rheumatology criteria [17]. These specimens represented moderate-to-severe OA. The Institutional Ethics Committee Board of the University of Montreal Hospital Research Centre (CRCHUM) approved the use of the human articular tissues and patients signed informed consent.

### Cell culture

Human chondrocytes were released from the cartilage as described [18], seeded at high density (10^5^/cm^2^), and cultured in Dulbecco’s modified Eagle’s medium (DMEM; Wisent, St-Bruno, QC, Canada) supplemented with 10 % heat-inactivated fetal bovine serum (FBS; PAA Laboratories Inc., Etobicoke, ON, Canada) and an antibiotic mixture (100 units/ml penicillin base and 100 μg/ml streptomycin base; Wisent) at 37 °C in a humidified atmosphere of 5 % CO_2_/95 % air. To preserve the chondrocyte phenotype, primary cells were used when comparing expression levels in normal and OA chondrocytes, and first-passage for experiments involving cultured cells. Of note, the cartilage and chondrocyte data are representative of independent assays and ‘n’ indicates the number of different patients.

An ER stress response was triggered by treating chondrocytes with Tm (500 ng/ml) or Tg (50 nM) for 20 hours in DMEM containing 10 % fetal bovine serum (FBS); under those conditions, neither Tg nor Tm affected cell viability (n = 8) as determined by the Thiazolyl Blue Tetrazolium Bromide (MTT, Sigma-Aldrich, Oakville, ON, Canada) assay as described [19] with modification [20]. The basal activation of the ER stress response in OA chondrocytes was determined by monitoring the three main sensors (ERN1, PERK, and ATF6B). OA chondrocytes were incubated without (basal control) and with Tg (50 nM) and Tm (500 ng/ml) for 20 minutes as controls for induced ER stress activation. The phosphorylation status of PERK was assessed by immunohistochemistry (IHC), the cleavage of ATF6B was assessed by Western blots using a specific antibody (see below), and the spliced forms of X-box binding protein 1 (XBP1) (target of ERN1) were monitored by polymerase chain reaction (PCR) using specific primers.

Gene regulation was performed by treating cells with growth factors and cytokines for 24 hours in DMEM containing 0.5 % FBS. Activin A (ActA), transforming growth factor beta (TGF-β), platelet-derived growth factor-BB (PDGF-BB), interleukin-4 (IL-4), IL-6, and IL-8 were used at a concentration of 10 ng/ml, IL-1β at 100 pg/ml, and tumour necrosis factor alpha (TNF-α) at 5 ng/ml.

### PCR array

Overall expression of 84 key UPR genes was determined with the RT2 Profiler PCR Arrays (PAHS-089Z; Qiagen, Venlo, The Netherlands) on an ABI 7900HT qPCR instrument (Applied Biosystems, Foster City, CA, USA) equipped with SDS 2.3 software, using RT2 SYBR Green ROX qPCR Master Mix (Qiagen). Total ribonucleic acid (RNA) from human chondrocytes was isolated and reverse-transcribed according to the manufacturer’s instructions. Data were analyzed by the 2^-ΔΔCt^ method. The relative quantity of complementary deoxyribonucleic acid (cDNA) was calculated using ribosomal protein large PO (RPLPO) as internal controls. All the PCR array data have been submitted to the Gene Expression Omnibus (GEO) public repository with the accession numbers GSE73749 (normal versus OA), GSE73748 (effect of Tm) and GSE73746 (effect of Tg).

### Quantification of mRNA and qPCR

Total RNA was extracted and messenger RNA (mRNA) levels quantified by real-time PCR (qPCR) with the SYBR Green PCR Master Mix (Qiagen) as described [21]. For total RNA quantification of expression between normal and OA chondrocytes, internal standards were added at known concentrations in the PCR reactions so as to give absolute numbers. The values of each sample were calculated as the ratio of the number of molecules of the target gene/number of molecules of the housekeeping gene. When evaluating the effect of a treatment, the expression level of each control was assigned an arbitrary value of 1, and the treated cells were evaluated as fold change over control and calculated as 2^-ΔΔCt^. Primer efficiencies for the genes under study were the same as those for the housekeeping gene, RPLPO. The sequences of the human specific primers used are listed in Additional file 1.

### Immunohistochemistry

The localization of ERN1, PERK, ATF6B, and CREB3L2 in normal and OA cartilage was determined by IHC as previously described [22]. In brief, the cartilage was fixed in TissuFix (Chaptec, Montreal, QC, Canada) and embedded in paraffin. Sections (5 μm) were deparaffinized in xylene followed by a graded series of alcohol washes. Sections were treated with 1 % hyaluronidase for 60 minutes at 37 °C (ERN1 only), 0.25 units/ml protease-free chondroitinase ABC in 0.1 M Tris acetate (Sigma-Aldrich), 0.3 % Triton for 30 minutes and 3 % H_2_O_2_ for 15 minutes at room temperature and proteinase K (20 μg/ml, 10 minutes at 37 °C for CREB3L2 only). 2 % goat serum (Vector Laboratories, Burlington, ON, Canada) was used for 45 minutes at room temperature to block unspecific staining prior to the addition of the primary antibodies.

The primary antibodies were diluted in goat serum and incubated overnight at 4 °C. Primary antibodies were all rabbit anti-human against ERN1 (monoclonal immunoglobulin G (IgG) 1/50; Cell Signaling Technology, Danvers, MA, USA), PERK (monoclonal IgG 1/100; Cell Signaling Technology), phosphorylated PERK (pPERK) (polyclonal IgG, 1/50, Santa Cruz Biotechnology, Santa Cruz, CA, USA), ATF6B (polyclonal IgG 1/100; Abcam, Cambridge, MA, USA), and CREB3L2 (polyclonal IgG 1/25; Santa Cruz Biotechnology). The tissues were washed in phosphate-buffered saline (PBS), then incubated with a secondary anti-rabbit antibody using a Vectastain ABC kit (Vector Laboratories) following the manufacturer’s instructions. The staining was developed with 3,3′-diaminobenzidine containing hydrogen peroxide, and sections were counterstained with eosin (0.03 % in 80 % ethanol). The eosin staining offers a good cell contrast allowing visualization of all the cells. Control procedures were performed by a substitution of the primary antibody with a nonspecific rabbit IgG (Santa Cruz Biotechnology) following the same experimental protocol; the results showed only background staining as illustrated in Additional file 2.

Antigen levels were quantified as described [22]. The total number of chondrocytes and the number of positively stained chondrocytes were quantitated separately for each zone of the cartilage. The final results are expressed as the percentage of positively stained chondrocytes.

### Gene silencing

Small interfering RNA (siRNA; Ambion-Life Technologies, Waltham, MA, USA) specific for the genes under study were transfected into the chondrocytes as described [21] for 48 hours (gene expression) or 72 hours (protein production). Cells either non-transfected or transfected with non-targeting (random) siRNAs (ON-TARGET*plus* Non-targeting siRNA; Dharmacon, Lafayette, CO, USA) served as controls and gave similar results. RNA was quantitated by qPCR and data normalized to the housekeeping gene RPLPO. When monitoring the effect of IL-1β on gene expression in the silenced chondrocytes, the cells were first transfected for 24 hours with the siRNAs, then IL-1β (100 pg/ml) was added and the cells incubated for another 24 hours in DMEM containing 0.5 % FBS.

### Western blotting

Total cellular proteins were extracted and processed for Western blotting as described [23]. For the silencing experiments, the primary antibodies were those used for the IHC assays with dilutions of 1/2000 (ERN1), 1/1000 (PERK and ATF6B) and 1/100 (CREB3L2). The secondary antibody was an anti-rabbit IgG (1/10,000; Pierce, Rockford, IL, USA). Glyceraldehyde-3-phosphate dehydrogenase (GAPDH) (monoclonal IgG, 1/20,000, Cell Signaling Technology) was used as the housekeeping (control) protein.

### Statistical analysis

Values are expressed as mean ± standard error of the mean (SEM). Statistical significance was assessed with the unpaired *t* test or a one-sample *t* test where appropriate; a *p* value ≤0.05 was considered significant.

## Results

### Several UPR genes are differentially expressed between normal and OA chondrocytes

The PCR array was first used to give an overview of the differential expression of the UPR genes in normal (n = 3) and OA (n = 3) chondrocytes. Table 1A lists the genes having a 1.3-fold expression change in OA compared to normal chondrocytes. Among the genes differentially expressed in OA are ERN1 and GRP78, which were downregulated. The expression of PERK was also downregulated to -1.2-fold, but the expression of ATF6 and ATF6B was unchanged.

**Table 1:** UPR genes having 1.3-fold change expression in human chondrocytes as determined by PCR array*

Increased expression		Decreased expression	
A) Osteoarthritic chondrocytes (n = 3) compared to normal chondrocytes (n = 3)			
SREBF2	4.71	CEBPB	-1.98
ERN2	1.81	UBE2G2	-1.87
ATXN3	1.61	HTRA4	-1.84
SCAP	1.57	NPLOC4	-1.73
SREBF1	1.52	**ERN1**	**-1.71**
PDIA3	1.49	DNAJB9	-1.60
ERO1L	1.48	**GRP78 (HSPA5)**	**-1.59**
HSPA4L	1.46	INSIG1	-1.51
DNAJB2	1.45	XBP1	-1.38
EDEM3	1.45	HTRA2	-1.32
SEC62	1.44		
NUCB1	1.40		
GANC	1.39		
SYVN1	1.35		
BAX	1.34		
INSIG2	1.33		
B) Osteoarthritic chondrocytes (n = 3) treated with thapsigargin (50 nM) compared to untreated (control) chondrocytes			
**GRP78 (HSPA5)**	**26.01**	PRKCSH	-4.37
**CHOP (DDIT3)**	**20.76**	SREBF1	-4.18
**HERPUD1**	**16.88**	MAPK10	-3.58
**DNAJB9**	**14.39**	HSPA2	-2.66
**ERO1LB**	**13.90**	PPIA	-2.57
MANF	8.69	HSPA4L	-2.18
PPP1R15A	6.98	FBXO6	-2.11
SEL1L	6.76	HSPA1L	-1.98
ERN1 (IRE1)	6.10	PFDN5	-1.91
SYVN1	5.99	MAPK9	-1.84
SERP1	4.98	GANC	-1.81
EDEM1	4.55	HTRA4	-1.77
SELS	4.49	CCT7	-1.75
PERK (EIF2AK3)	4.15	HSPA1B	-1.71
DERL2	3.90	INSIG1	-1.70
DNAJC3	3.67	NPLOC4	-1.64
PDIA3	2.81	BAX	-1.62
SEC63	2.54	HSPA4	-1.58
DNAJC10	2.48	DNAJB2	-1.57
DERL1	2.27	TOR1A	-1.55
ATF4	2.17	UBE2G2	-1.51
CALR	2.13	MBTPS1	-1.45
XBP1	1.98	GANAB	-1.43
ERP44	1.89	SREBF2	-1.39
ERO1L	1.75	ERN2	-1.36
VCP	1.72	HSPH1	-1.35
UGGT1	1.60	AMFR	-1.35
ATF6	1.54	SIL1	-1.31
OS9	1.52	ATF6B	-1.30
CANX	1.52		
RPN1	1.42		
RNF5	1.34		
C) Osteoarthritic chondrocytes (n = 3) treated with tunicamycin (500 ng/ml) compared to untreated (control) chondrocytes			
**GRP78 (HSPA5)**	**21.89**	SREBF1	-3.79
**CHOP (DDIT3)**	**15.69**	INSIG1	-3.17
**HERPUD1**	**11.67**	HSPA2	-2.77
**ERO1LB**	**10.34**	PPIA	-2.27
**DNAJB9**	**9.44**	HSPA4L	-2.03
MANF	6.76	PRKCSH	-1.97
SEL1L	5.66	HTRA4	-1.95
ERN1 (IRE1)	4.57	SREBF2	-1.94
SERP1	3.94	FBXO6	-1.91
PPP1R15A	3.75	NPLOC4	-1.74
DNAJC3	3.67	MAPK10	-1.70
SYVN1	3.58	MAPK9	-1.63
EDEM1	3.53	CCT7	-1.62
SELS	3.34	PFDN5	-1.61
DERL2	3.04	HSPA1B	-1.60
PERK (EIF2AK3)	2.27	HSPH1	-1.59
ATF4	2.20	GANC	-1.57
PDIA3	2.09	ERN2	-1.47
SEC63	2.03	HSPA4	-1.46
DNAJC10	2.01	TOR1A	-1.46
CALR	1.86	INSIG2	-1.41
DERL1	1.80	UDF1L	-1.40
ERP44	1.59	TCP1	-1.39
UGGT1	1.58	MBTPS1	-1.35
XBP1	1.47		
ATF6	1.45		
CANX	1.37		

*The RPLPO gene was used as the housekeeping reference gene. Genes in bold are those cited in the text

**Fig. 1:**
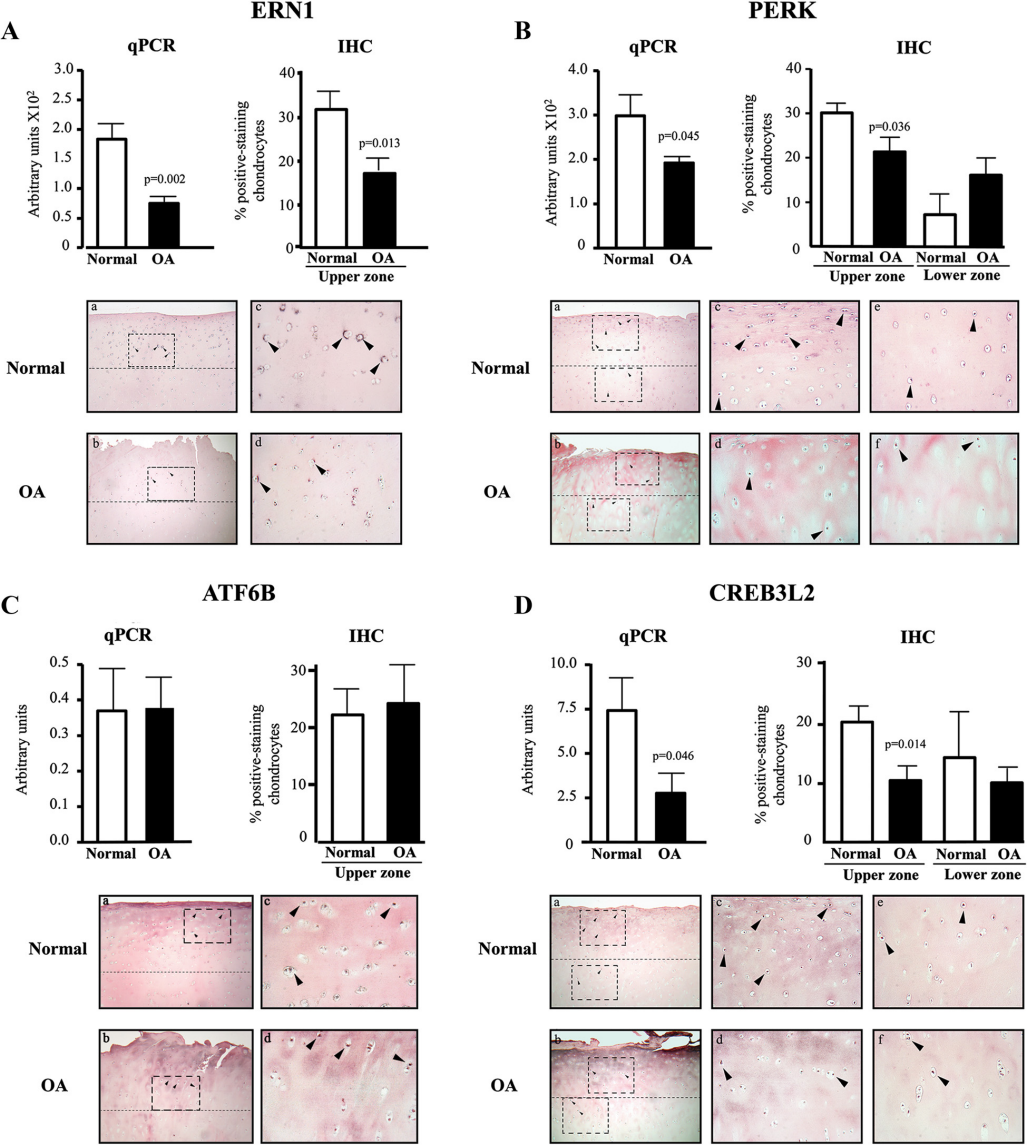
Expression/production of UPR genes in normal and osteoarthritic (OA) human chondrocytes and cartilage. mRNA levels (qPCR, normal [n = 6)] and OA [n = 6]) and protein production (immunohistochemistry [IHC], normal [n = 7] and OA [n = 7]) of (**a**) ERN1, (**b**) PERK, (**c**) ATF6B, and (**d**) CREB3L2. Illustrated are representative images of cartilage IHC. Data are expressed as mean ± SEM, and *p* values assessed by the unpaired *t* test comparing OA to normal chondrocytes. Magnification × 63 (a, b) and × 250 (c and d, *upper cartilage zone*; e and f, *lower zone*); *boxes* indicate where the magnifications were taken, and *arrows* indicate positively stained chondrocytes

Validation by qPCR (Fig. 1) was performed for the representatives of the three main branches of the ER stress response, ERN1, PERK, and ATF6B, a member of the ATF6 family. Data include CREB3L2, also a member of the ATF6 family [10]. Data confirm that ERN1 (Fig. 1a) and PERK (Fig. 1b) expression was significantly decreased in OA (n = 6) compared to normal (n = 6) chondrocytes. ATF6B (Fig. 1c) was similarly expressed in normal and OA chondrocytes, but CREB3L2 expression (Fig. 1d) was significantly decreased in OA chondrocytes.

Next, we looked at whether the expression of ERN1, PERK, ATF6B, and CREB3L2 was reflected in protein production by performing IHC directly on cartilage to determine both the amount and location in the cartilage of the proteins tested. The number of chondrocytes that stained positive for ERN1 (Fig. 1a) and ATF6B (Fig. 1c), was less than 5 % in the lower zone of the cartilage, whereas PERK (Fig. 1b) and CREB3L2 (Fig. 1d) were detected in both the upper and lower zones of the cartilage, although at a reduced level in the lower zone compared to the upper zone. ERN1, PERK, and CREB3L2 production was significantly reduced in the upper zone of the OA cartilage, but ATF6B was similar in both normal and OA cartilage. The IHC data (from cartilage) mirrored the qPCR data (from isolated chondrocytes), indicating that the expression/production of the representatives of the three main branches of the ER stress response are downregulated in OA chondrocytes/cartilage.

### Basal activation status of the ER stress response in OA chondrocytes

**Fig. 2:**
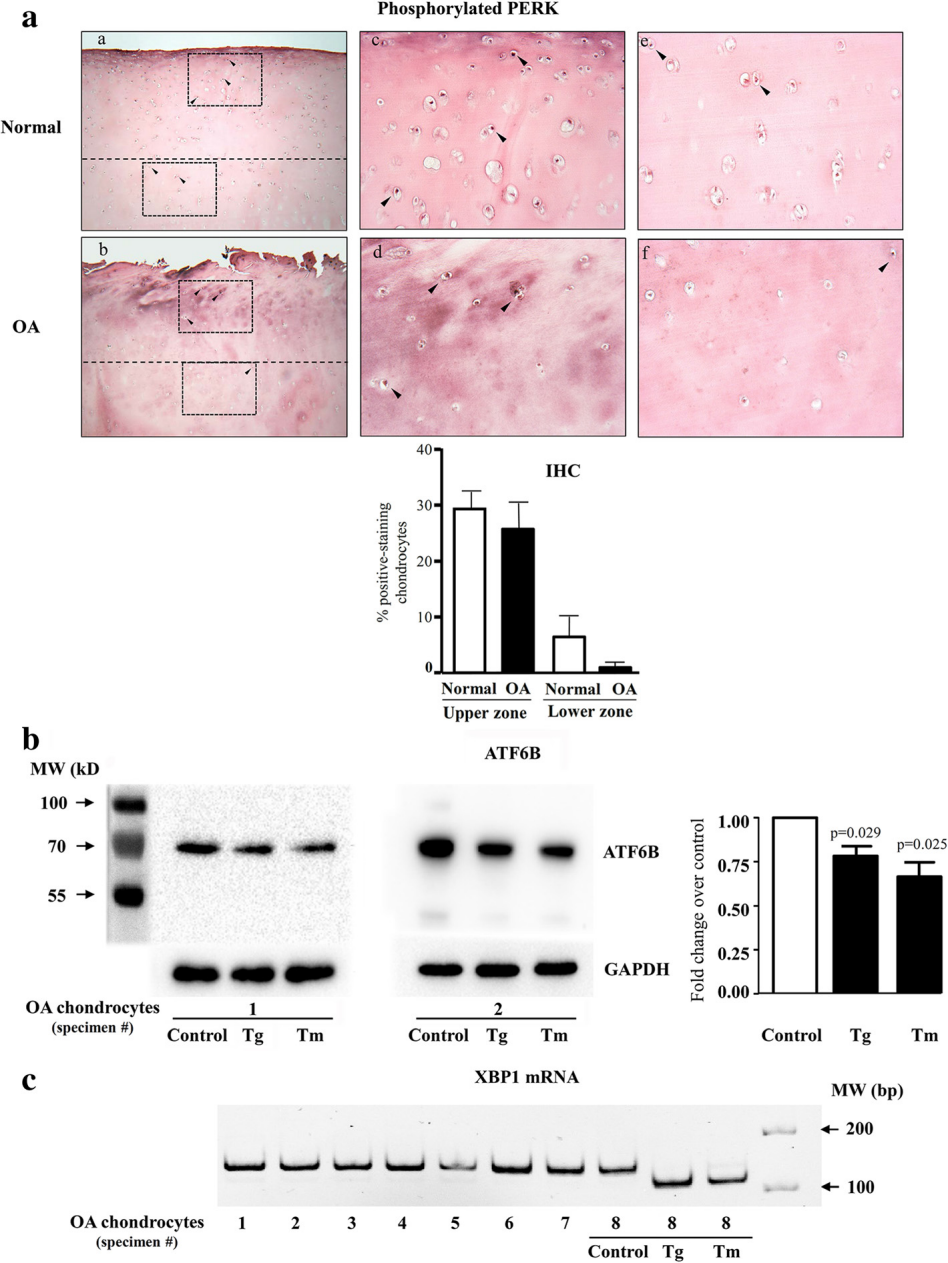
Basal activation status of the ER stress sensors in osteoarthritic (OA) chondrocytes. **a** The phosphorylation of PERK was determined by immunohistochemistry (IHC) in normal (n = 7) and OA (n = 7) cartilage. Illustrated are representative images of cartilage IHC and a graph of the data expressed as mean ± SEM. Statistical significance assessed by the unpaired *t* test comparing OA to normal chondrocytes showed no difference. Magnification × 63 (a, b) and × 250 (c and d, *upper cartilage zone*; e and f, *lower zone*); *boxes* indicate where the magnifications were taken, and *arrows*, the positively stained chondrocytes. **b** The uncleaved form (approximately 75 kDa) of ATF6B in OA chondrocytes (n = 6) treated or not (control) for 20 minutes with thapsigargin (Tg; 50 nM) and tunicamycin (Tm; 500 ng/ml) assessed by Western blot using an antibody that recognizes the C-terminus of the protein. Illustrated are representative Western blots with GAPDH as loading control and a graph of the densitometry analysis of the uncleaved ATF6B. Data are expressed as mean ± SEM, and *p* values assessed by the unpaired *t* test comparing OA to normal chondrocytes. **c** The presence of the spliced form of XBP1 mRNA was determined by PCR followed by gel electrophoresis (n = 8). In addition, OA chondrocyte sample #8 treated for 20 minutes with Tg (50 nM) and Tm (500 ng/ml) is shown as control for the ER stress activation

The basal activation status of the three main branches of the ER stress response (phosphorylation of PERK, cleavage of ATF6B and splicing of XBP1, a target of ERN1) was monitored in untreated OA cells. The presence of phosphorylated PERK was determined by IHC in normal and OA cartilage, the ATF6B total protein by Western blot in OA chondrocytes, and XBP1 splicing by PCR in OA chondrocytes. Although data (Fig. 2a) showed that the levels of phosphorylated PERK were slightly decreased in OA compared to normal cartilage, the difference did not reach statistical significance.

When activated by ER stress, ATF6B (approximately 75 kDa) is cleaved into fragments, one of which acts as a transcription factor following its transfer to the nucleus. The cleavage of ATF6B was assayed by Western blot with an antibody recognizing the C-terminus of the protein. Samples treated with Tg (50 nM) and Tm (500 ng/ml), agents well known to induce an ER stress response (I-UPR conditions), were included as controls for the cleavage of the protein. As illustrated in Fig. 2b, Tg or Tm treatments significantly reduced the levels of the uncleaved ATF6B in OA chondrocytes, suggesting that if activated, it is not at its maximal levels. Although the antibody could in theory also detect the small cleaved fragment, under our experimental conditions this fragment was barely detectable, possibly due to further degradation.

The presence of the XBP1-spliced mRNA was determined by PCR with primers that amplify both the unspliced (amplification product of 145 bp) and spliced (amplification product of 119 bp) forms. Samples treated with Tg (50 nM) and Tm (500 ng/ml) were also included as controls of XBP1 mRNA splicing. Data (Fig. 2c) show that the unspliced mRNA was the only XBP1 mRNA species detected in all the untreated OA cells. This is in contrast to the Tg- and Tm-treated OA chondrocytes, which showed only the activated (spliced) XBP1 mRNA species.

Taken together, these results indicate that the ER stress response is not globally or fully activated in OA chondrocytes.

### Regulation of UPR genes under I-UPR conditions in normal and OA chondrocytes

Next, we assessed whether the decreased expression of some UPR genes in OA chondrocytes affected the induced ER stress response. To this end, OA chondrocytes (n = 3) were incubated with Tg (50 nM) and Tm (500 ng/ml). Table 1B and C list the UPR genes having 1.3-fold change expression for Tg and Tm treatments, respectively. The greatest increased expression was observed with GRP78 (target gene of ERN1 and ATF6) and DNA damage-inducible transcript 3 (DDIT3) (CHOP, target gene of PERK) [6, 7]. Increased expression was also noted with the homocysteine-inducible, endoplasmic reticulum stress-inducible, ubiquitin-like domain member 1 (HERPUD1), DnaJ (Hsp40) homolog, subfamily B, member 9 (DNAJB9), and ERO1-like beta (S. Cerevisiae) (ERO1LB) genes, which are the targets of CREB3L2 [11]. These results indicate that the OA chondrocytes could respond strongly to acute ER stress. Validation of OA chondrocytes (n = 6–12) by qPCR (Table 2) also resulted in significantly increased expression of GRP78, CHOP, DNAJB9, ERN1, and PERK; ATF6B, CREB3L2, and sterol regulatory element-binding protein 2 (SREBP2) (with Tg) expression was not significantly affected. Treatment of normal chondrocytes (n = 4) with Tg and Tm showed a similar level of response to that observed in OA chondrocytes (Table 2) and data revealed no significant differences between them. These results indicate that ERN1 and PERK, despite their decreased expression in OA chondrocytes (Fig. 1), could be activated and trigger a strong response.

**Table 2:** Expression of UPR genes in response to treatment with thapsigargin and tunicamycin in human chondrocytes

	Tg				Tm			
	Normal	*p* value	OA	*p* value	Normal	*p* value	OA	*p* value
ERN1	5.29 ± 1.49	0.063	7.15 ± 0.61	≤0.001	3.76 ± 1.12	0.089	4.12 ± 0.36	≤0.001
PERK	3.54 ± 0.34	0.005	2.52 ± 0.21	≤0.001	2.18 ± 0.48	0.089	1.59 ± 0.12	0.006
ATF6B	1.05 ± 0.06	0.446	0.82 ± 0.08	0.074	1.07 ± 0.30	0.837	0.86 ± 0.12	0.325
CREB3L2	1.15 ± 0.26	0.606	1.38 ± 0.34	0.307	1.15 ± 0.20	0.502	1.20 ± 0.16	0.271
GRP78	38.54 ± 6.27	0.009	35.68 ± 4.45	≤0.001	19.18 ± 2.40	0.005	16.74 ± 2.31	≤0.001
CHOP	22.08 ± 6.72	0.052	25.64 ± 6.49	0.012	13.66 ± 3.06	0.025	6.34 ± 1.66	0.023
DNAJB9	15.04 ± 4.37	0.049	12.33 ± 1.77	≤0.001	9.56 ± 2.86	0.009	7.76 ± 0.75	≤0.001
SREBP2	0.79 ± 0.11	0.165	0.84 ± 0.11	0.200	0.51 ± 0.09	0.013	0.68 ± 0.09	0.015

The normal (n = 4) and osteoarthritic (OA; n = 6–12) chondrocytes were treated for 20 hours with thapsigargin (Tg; 50 nM) and tunicamycin (Tm; 500 ng/ml) and processed for qPCR. Each untreated control was assigned an arbitrary value of 1. The effect of each treatment was evaluated as fold change over control and expressed as mean values ± SEM. *p* values were assessed by the one-sample *t* test, comparing the treated chondrocytes to the untreated controls (arbitrary value of 1); values ≤0.05 were considered significant. Comparison between normal and OA was assessed by the Mann-Whitney test; data revealed no significant differences (*p* values not shown)

### Regulation and role of UPR genes under H-UPR conditions

As the role of UPR proteins under I-UPR conditions has already been investigated [24–26], we further explored the regulation and role of the UPR genes under H-UPR conditions. To this end, we hypothesized that some of those proteins played a role under H-UPR conditions, different from those under I-UPR. We first looked at factors that could be responsible, under homeostatic conditions, for the downregulation of ERN1, PERK, and CREB3L2 in OA chondrocytes and secondly determined their potential role in regulation of genes involved in OA pathology.

#### ERN1, PERK, and CREB3L2 expression is downregulated by PDGF-BB and IL-6 in OA chondrocytes

**Fig. 3:**
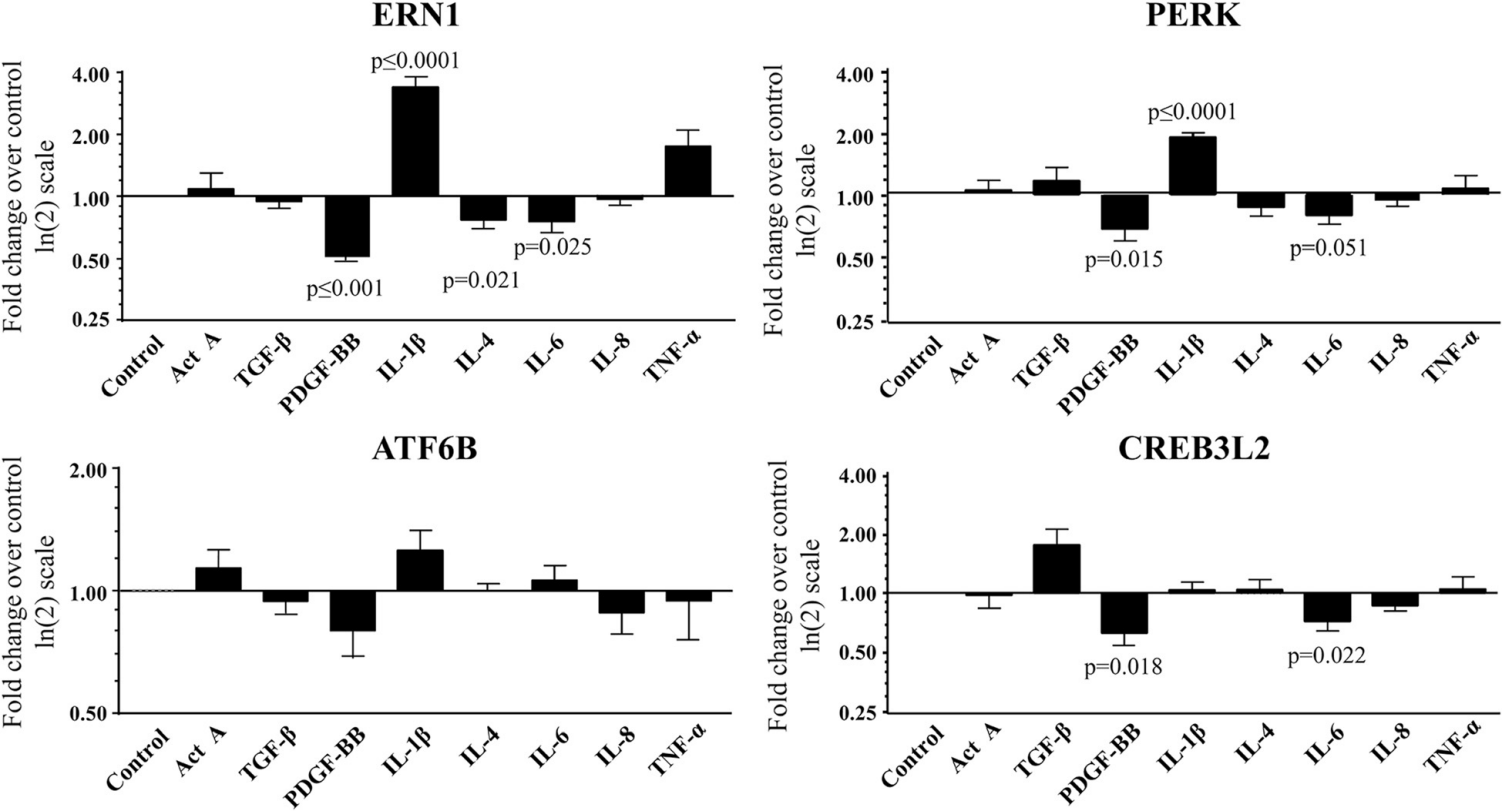
Regulation of UPR genes by cytokines/growth factors in human osteoarthritic chondrocytes under H-UPR conditions. Chondrocytes (n = 5–7) were treated with the following factors: Activin A (ActA), TGF-β, PDGF-BB, IL-4, IL-6, and IL-8 (all at 10 ng/ml), IL-1β (100 pg/ml), and TNF-α (5 ng/ml) and processed for RNA extraction and qPCR. Each control (untreated cells) was assigned an arbitrary value of 1 and the effect of the treatment evaluated as fold change over control. Data are expressed as mean ± SEM, and *p* values assessed by the one-sample *t* test, comparing the treated chondrocytes to the control

OA chondrocytes (n = 5–7) were treated with cytokines and growth factors known to be associated with cartilage pathobiology and their effect on ERN1, PERK, ATF6B, and CREB3L2 was assessed. Data (Fig. 3) revealed that IL-1β significantly increased ERN1 and PERK expression. Interestingly, PDGF-BB and IL-6 significantly decreased ERN1, PERK, and CREB3L2, while IL-4 significantly decreased only ERN1 gene expression. ATF6B, unlike CREB3L2, was not significantly affected by the factors studied. It is interesting to note that members of the ATF6 family (ATF6, ATF6B, and CREB3L2) are not only differentially expressed in OA chondrocytes (Fig. 1), but are also differentially regulated (Fig. 3). Thus, although members of the ATF6 family share similar functions as effectors of ER stress, CREB3L2 may be the factor of choice in human chondrocytes for fine-tuning the regulation of ER stress.

We have shown that the expression of ERN1 and PERK was decreased in OA cells and that IL-1β increased PERK and ERN1 expression. As IL-1β is known to be increased in OA [27], we further determined whether the presence of PDGF-BB and IL-6 could interfere with the upregulation of ERN1 and PERK by IL-1β. To this end, OA chondrocytes were monitored for the expression of IL-1β following PDGF-BB and IL-6 treatments. Data show that treatment of OA chondrocytes with PDGF-BB resulted in a significantly decreased expression of IL-1β (n = 6, mean fold change over control ± SEM 0.50 ± 0.13, *p* = 0.013 [one-sample *t* test]), but that treatment with IL-6 did not significantly affect IL-1β expression (n = 6, 1.8 ± 1.0, *p* = 0.437). These results show that the presence of PDGF-BB can interfere with the expression levels of IL-1β and ultimately those of ERN1 and PERK.

#### ERN1, PERK, ATF6B, and CREB3L2 regulate collagen and protease gene expression

**Fig. 4:**
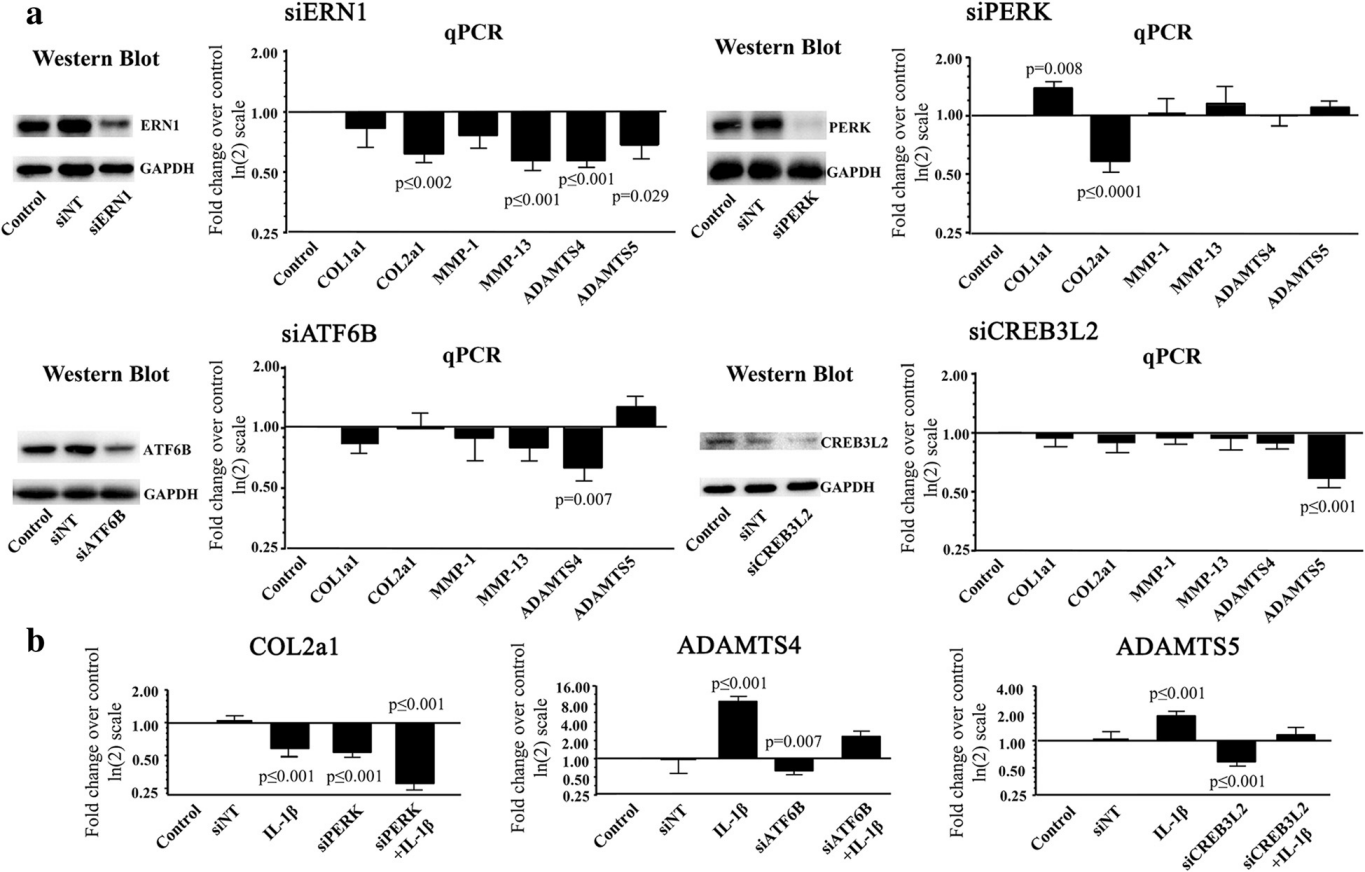
Effect of silencing UPR genes under H-UPR conditions in human osteoarthritic chondrocytes. **a** Silencing (si) ERN1, PERK, ATF6B, and CREB3L2 (Western blots), and the resulting expression of COL1a1, COL2a1, MMP-1, MMP-13, ADAMTS4, and ADAMTS5 (qPCR) (n = 7–10). The non-targeting or random siRNA (siNT) values were calculated for each primer as fold change over the control, which was assigned an arbitrary value of 1, and the mean ± SEM was 1.02 ± 0.03 for all the primers grouped. **b** Effect of IL-1β (100 pg/ml) with or without (control) siPERK, siATF6B, and siCREB3L2 on COL2a1, ADAMTS4, and ADAMTS5 levels (n = 5–7). Each control was assigned an arbitrary value of 1 and the effect of the treatment evaluated as fold change over control. Also represented are the siNTs for COL2a1, ADAMTS4, and ADAMTS5 respectively, showing values similar to those of the control. Data are expressed as mean ± SEM, and *p* values were assessed by the one-sample *t* test, comparing the treated chondrocytes to the control

We next determined whether ERN1, PERK, ATF6B, and CREB3L2 affected the expression of genes involved in OA pathology. The genes of interest were silenced with specific siRNAs and the effect assessed on the expression of types I (COL1a1) and II (COL2a1) collagens, matrix metalloproteinases (MMP)-1 and MMP-13, and a disintegrin and metalloproteinase with thrombospondin motifs (ADAMTS)4 and ADAMTS5. As illustrated in Fig. 4a, silencing ERN1 induced a decreased expression of COL2a1, MMP-13, ADAMTS4, and ADAMTS5, ATF6B significantly decreased expression of ADAMTS4, and CREB3L2 the expression of ADAMTS5. Interestingly, silencing PERK resulted in a significant increase in the expression of COL1a1 and a significant decrease in COL2a1 gene expression.

Thus, under H-UPR conditions, increasing PERK may contribute to an increase in type II collagen and a decrease in type I collagen expression, whereas increasing ERN1, ATF6B, and CREB3L2 could have detrimental effects as they would upregulate MMP-13, ADAMTS4, and ADAMTS5, known catabolic factors involved in cartilage destruction.

As IL-1β is an important factor in the regulation of collagen and protease genes, we examined whether the decreased expression of ERN1, PERK, ATF6B, or CREB3L2 affected its action. IL-1β significantly reduced COL2a1 and increased MMP-13 (data not shown), ADAMTS4, and ADAMTS5 expression (Fig. 4b), but did not affect COL1a1 expression (data not shown). Silencing ERN1 did not affect the stimulatory effect of IL-1β on MMP-13 and ADAMTS4 or its inhibitory effect on COL2a1 (data not shown). IL-1β did not maximally stimulate ADAMTS4 in the silenced ATF6B or ADAMTS5 in the silenced CREB3L2 chondrocytes (Fig. 4b). IL-1β did not affect COL1a1 in PERK-silenced chondrocytes (data not shown), but significantly reduced COL2a1 (Fig. 4b). Thus, a lower expression of PERK has a twofold effect: it reduces COL2a1 expression and, in the presence of IL-1β such as occurs in OA, the COL2a1 expression is further reduced.

## Discussion

The event that triggers the initiation of OA is still a matter of intense research. OA is a multifactorial process with several factors contributing to the degradation of the cartilage. In this work, we examined whether OA was characterized by an overall activation of the ER stress response. We first looked at the global expression of UPR genes in human OA chondrocytes, the activation status of the ER stress sensors, and the response of OA cells under induced (I)-UPR conditions. We found that despite the decreased expression/production of several UPR genes in OA, the global induced ER stress response is similar to that of normal chondrocytes, and that the three main sensors of the ER stress were not activated or fully activated in OA cells. Based on these data, OA is not likely the result of an overall activation of the ER stress pathways. Interestingly, data revealed that the reduced expression of the UPR genes could influence the progression of OA under homeostatic (H)-UPR conditions, in which ERN1, PERK, and CREB3L2 can act as regulators of some collagens/MMPs. As the decrease in PERK favours a reduced expression of type II collagen (COL2a1) and increased type I collagen, these findings lead us to propose PERK as a potential target for OA therapeutic strategies. However, further studies are needed to confirm this finding.

Our finding that OA cells are not characterized by an increased activation of the ER stress response is in contrast to previous reports suggesting that GRP78 and phosphorylated PERK increased with the severity of cartilage degeneration [15, 16]. However, Takada et al [15] only looked at OA cartilage and indeed detected activated pPERK in this tissue, as we found in our OA samples. The amount of pPERK in our OA samples, however, was not greater than that found in the normal cartilage. Takada et al [15] also investigated the presence, by gel electrophoresis, of the XBP1 spliced form. They looked at OA human cartilage samples but from different regions named mild, moderate, and severe degeneration from the same knee. They showed that the spliced form of XBP1 was barely or not at all detectable in the mild and severe regions of the cartilage. Although present in moderate OA cartilage, it was less abundant than the unspliced form, indicating that the XBP1 spliced form was not the major form expressed. In our study, we used cartilage samples taken from the whole OA knee to obtain a global picture of XBP1 activity and did not detect the spliced form.

We have shown that PDGF-BB and IL-6 will add to the degenerative process of OA through the downregulation of ERN1 and PERK, which, in turn, will affect collagen expression. This finding adds to the known effects of PDGF-BB on chondrocyte proliferation, differentiation and proteoglycan production [28], and downregulation of the IL-1β-induced nuclear factor kappa B (NF-kB) signalling [29]. Data also revealed a new role for IL-6, known as an independent predictor of the appearance of knee OA, for its association with local inflammation [30, 31], and for its capacity to downregulate the expression of type II collagen [32]. As we have shown that IL-6 decreases ERN1 and PERK expression and that a decrease in these two proteins leads to a decreased expression of type II collagen, these data illustrate the versatility of action of this cytokine on type II collagen expression in OA chondrocytes.

The finding that IL-1β upregulated ERN1 and PERK was unexpected, considering that their expression was decreased in OA chondrocytes and that IL-1β is known to be increased in diseased cartilage [27]. It is possible that the increased presence of PDGF-BB in the OA cartilage [33] would have a threefold effect: a decreased expression of ERN1 and PERK by directly reducing their expression, a decreased IL-1β expression as our results have shown, and an interference with the stimulatory effect of IL-1β on those genes [29].

An emerging finding is that, under homeostasis, the decreased expression of the UPR genes ERN1, PERK, ATF6B, and CREB3L2 in human chondrocytes will affect the regulation of some collagens/MMPs. As reported in other systems, PERK and ERN1 play dual roles depending on whether they are activated by physiological factors or by ER stress and the cellular response will differ depending on the stimuli. For instance, Lipson et al [12] reported that activation of ERN1 in pancreatic β cells transiently exposed to high glucose resulted in an increased insulin biosynthesis not involving the activation of the ER stress response, but that a chronic exposure to high glucose triggered the ER stress response and the suppression of the insulin gene expression. Another example of the role of UPR genes in the absence of ER stress has been shown in endothelial cells, where vascular endothelial growth factor (VEGF) signals through ATF6 and PERK via the mammalian target of rapamycin complex 1 (mTORC1) complex [13]. In the present study, we identify ERN1 and PERK as regulators of collagen and protease expression in OA chondrocytes under H-UPR conditions. The regulation of collagen synthesis in chondrocytes is crucial as the type of collagen produced influences the quality of the cartilage matrix. In OA, there is a loss of the structural collagen, the type II, and an increase in type I collagen known to weaken the cartilage, which undermine the integrity of the extracellular cartilage matrix, in turn affecting the cartilage properties. Our data showing that a decreased expression of ERN1 and PERK in OA chondrocytes could induce a decreased expression of type II collagen and, in the case of PERK, an increased expression of type I collagen, are of great importance as this would favour a microenvironment change in the extracellular matrix and contribute to the degeneration process. In turn, increasing the molecules ERN1 and PERK in OA cartilage could be foreseen as having therapeutic potential. However, as ERN1, ATF6B, and CREB3L2 affect proteases involved in cartilage degradation, their usage during OA could be detrimental. In contrast, an increase in PERK, in addition to acting in a reverse manner on both types of collagen, could also counteract the inhibitory effect of IL-1β on type II collagen.

## Conclusions

In conclusion, we showed that the level of ER stress is not significantly elevated in OA chondrocytes. Furthermore, under I-UPR conditions, OA chondrocytes respond strongly to ER stress despite the decreased expression of several ER stress genes. Importantly, and for the first time, this study reveals new roles for ERN1, PERK, ATF6B, and CREB3L2 in cartilage under H-UPR conditions as regulators of collagen and/or protease genes, and identifies PERK as a potential target for new therapeutic strategies for OA as increasing PERK may lead to an increase in type II collagen and a decrease in type I collagen expression and, in so doing, could restore to some extent homeostatic conditions in OA cartilage.

## Abbreviations

ActA, Activin A; ADAMTS, a disintegrin and metalloproteinase with thrombospondin motifs; ATF6, activating transcription factor 6; cDNA, complementary DNA; CREB3L2, CAMP-responsive element-binding protein 3-like 2; DDIT3, DNA damage-inducible transcript 3; DMEM, Dulbecco’s modified Eagle’s medium; DNA, deoxyribonucleic acid; DNAJB9, DnaJ (Hsp40) homolog, subfamily B, member 9; ER, endoplasmic reticulum; ERN 1, endoplasmic reticulum to nucleus 1; ERO1LB, ERO1-like beta (S. Cerevisiae); FBS, fetal bovine serum; GAPDH, glyceraldehyde-3-phosphate dehydrogenase; GRP78, glucose-regulated protein 78; HERPUD1, homocysteine-inducible, endoplasmic reticulum stress-inducible, ubiquitin-like domain member 1; H-UPR, homeostatic unfolded protein response; IgG, immunoglobulin G; IHC, immunohistochemistry; IL, interleukin; I-UPR, induced unfolded protein response; MMP, matrix metalloproteinases; mRNA, messenger RNA; mTORC1, mammalian target of rapamycin complex 1; NF-kB, nuclear factor kappa B; NRF2, nuclear factor erythroid 2; OA, osteoarthritis; OASIS, old astrocyte specifically induced substance; PCR, polymerase chain reaction; PDGF-BB, platelet-derived growth factor-BB; PERK, PRKR-like endoplasmic reticulum kinase; pPERK, phosphorylated PERK; qPCR, quantitative real-time PCR; RNA, ribonucleic acid; RPLPO, ribosomal protein large PO; SD, standard deviation; SEM, standard error of mean; siRNA, small interfering RNA; SREBP2, sterol regulatory element-binding protein 2; Tg, thapsigargin; TGF-β, transforming growth factor beta; Tm, tunicamycin; TNF-α, tumour necrosis factor alpha; UPR, unfolded protein response; VEGF, vascular endothelial growth factor; XBP1, X-box binding protein 1

## Additional files

Additional file 1: Sequences of the human gene-specific primers used for qPCR. (PDF 25 kb) Additional file 2: Immunohistochemistry negative controls. (PDF 122 kb)
