# Supplementary material for: The unfolded protein response genes in human osteoarthritic chondrocytes: PERK emerges as a potential therapeutic target
Source: Arthritis Res Ther. 2016 Jul 19;18:172. doi: 10.1186/s13075-016-1070-6 (PMC4952234; doi:10.1186/s13075-016-1070-6)
Supplement: Additional file 1: — Sequences of the human gene-specific primers used for qPCR. (PDF 25 kb) [file 13075_2016_1070_MOESM1_ESM.pdf]

Additional File 1

The unfolded protein response genes in human osteoarthritic chondrocytes: PERK emerges as a potential therapeutic target

Li YH, Tardif G, Hum D, Kapoor M, Fahmi H, Pelletier JP, Martel-Pelletier J

Sequences of the human gene-specific primers used for qPCR

**Gene**<br>(GenBank accession number)	**Sequence**
ADAMTS4	5’- CTGTGATGGCTCATGTGGAT (S)
(NM_005099)	5’- GGGAAAGTCACAGGCAGATG (AS)
ADAMTS5	5’- GGCATCATTCATGTGACAC (S)
(BC093775.1)	5’- GCATCGTAGGTCTGTCCTG (AS)
ATF4	5’- CTTCCTGAGCAGCGAGGTG (S)
(NM_001675.3)	5’- TCTCCAACATCCAATCTGTCC (AS)
ATF6B	5’- GAGTCATCGCGTCTCTCCAC (S)
(NM_ 004381)	5’- GGCCTCAGAGTTGACGGAAG (AS)
CHOP (DDIT3)	5’- AAGGCACTGAGCGTATCATGT (S)
(NM_001195053)	5’- TGAAGATACACTTCCTTCTTGAACA (AS)
COL1a1	5’- GCAACATGGAGACTGGTGAG (S)
(AF017178.2)	5’- GCTGTTCTTGCAGTGGTAGG (AS)
COL2a1	5’- CACACTCAAGTCCCTCAACAA (S)
(NM_ 001844)	5’- AGTAGTCTCCACTCTTCCACTC (AS)
CREB3L2	5’- CTCTCATCCAGGCTGAGCAC (S)
(NM_194071)	5’- CTGTAACTGGCTCTGTCTTG (AS)
DNAJB9	5’- AAGGCCTTTCACAAGTTGGC (S)
(NM_012328)	5’- ACGCTTCTTGGATCCAGTGTT (AS)
ERN1	5’- AGAGAAGCAGCAGACTTTGTC (S)
(NM_ 001433)	5’- GTTTTGGTGTCGTACATGGTGA (AS)
GAPDH	5’- CAGAACATCATCCCTGCCTCT (S)
(NM_002046.3)	5’- GCTTGACAAAGTGGTCGTTGAG (AS)
GRP78 (HSPA5)	5’- GGATCATCAACGAGCCTACG (S)
(NM_ 005347)	5’- CACCCAGGTCAAACACCAG (AS)
IL-1β	5’- CCTGTACGATCACTGAACTG (S)
(BC008678.1)	5’- TGGGCAGACTCAAATTCCAG (AS)
IL-6	5’- CACCTCTTCAGAACGAATTG (S)
(M14584.1)	5’- CTAGGTATACCTCAAACTCC (AS)
MMP-1	5’- CTGAAAGTGACTGGGAAACC (S)
(NM_002421.3)	5’- AGAGTTGTCCCGATGATCTC (AS)
MMP-13	5’- CTTAGAGGTGACTGGCAAAC (S)
(NM_002427.3)	5’- GCCCATCAAATGGGTAGAAG (AS)
NRF2	5’- CCAGTGGATCTGCCAACTAC (S)
(NM_ 006164.4)	5’- AGGTGACTGAGCCTGATTAG (AS)
PDGF-BB	5’- CTCGTGGAAGAAGGAGCCTG (S)
(NM_002608.3)	5’- GCGTTGGTGCGGTCTATGAG (AS)
PERK (EIF2AK3)	5’- GTCCGGAACCAGACGATGAG (S)

(NM_ 004836)	5’- GGCTGGATGACACCAAGGAA (AS)
RPLPO	5’- GGCAGCATCTACAACCCTGA (S)
(NM_ 001002.3)	5’- CCAGGACTCGTTTGTACCCG (AS)
XBP1	5’- CCTGGTTGCTGAAGAGGAGG (S)
(AB076383.1/AB076384.1)	5’- CCATGGGGAGATGTTCTGGAG (AS)

S: sense; AS: antisense
