# Supplementary material for: The unfolded protein response genes in human osteoarthritic chondrocytes: PERK emerges as a potential therapeutic target
Source: Arthritis Res Ther. 2016 Jul 19;18:172. doi: 10.1186/s13075-016-1070-6 (PMC4952234; doi:10.1186/s13075-016-1070-6)

Additional File 2

The unfolded protein response genes in human osteoarthritic chondrocytes: PERK emerges as a potential therapeutic target

Li YH, Tardif G, Hum D, Kapoor M, Fahmi H, Pelletier JP, Martel-Pelletier J

Immunohistochemistry negative controls. Control procedures for immunohistochemistry (IHC) were performed by substituting the primary antibody with a nonspecific rabbit IgG. Illustrated are representative IHC of normal cartilage for ERN1, PERK, ATF6B, CREB3L2 and pPERK (n=7). The controls showed only background staining. Magnification X63.

ERN1

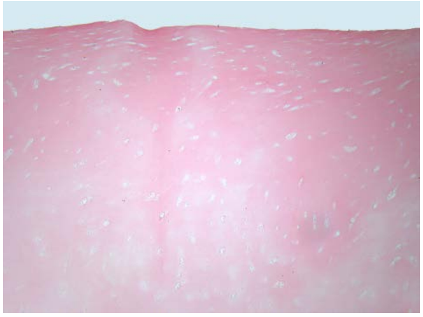

PERK

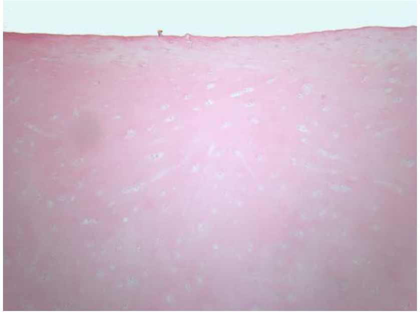

ATF6B

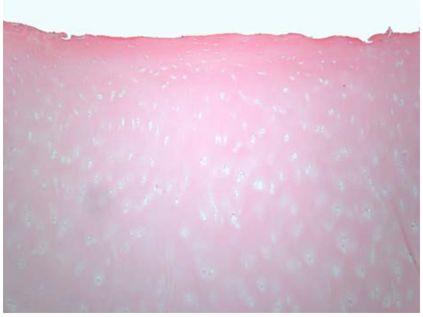

CREB3L2

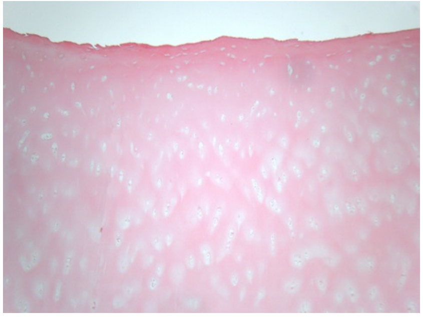

pPERK

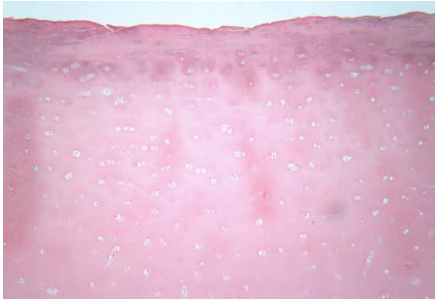

Supplement: Additional file 2: — Immunohistochemistry negative controls. (PDF 122 kb) [file 13075_2016_1070_MOESM2_ESM.pdf]
